# Combination of conventional immunohistochemistry and qRT-PCR to detect ALK rearrangement

**DOI:** 10.1186/1746-1596-9-3

**Published:** 2014-01-14

**Authors:** Ling Shan, Fang Lian, Lei Guo, Xin Yang, Jianming Ying, Dongmei Lin

**Affiliations:** 1 Department of Pathology, Cancer Institute & Hospital, Chinese Academy of Medical Sciences, Peking Union Medical College, Beijing, 100021, China

**Keywords:** Immunohistochemistry, Fluorescence *in situ* hybridization, qRT-PCR, ALK rearrangement, D5F3 antibody, Lung adenocarcinoma

## Abstract

**Background:**

Compared with FISH and qRT-PCR analyses, immunohistochemistry (IHC) is the preferred screening test in most pathology practices for ALK-rearrangement detection. With 100% sensitivity and 98% specificity, the VENTANA ALK (D5F3) IHC assay has been approved in the EU and some Asian countries for ALK-rearrangement detection. However, an automated Ventana IHC platform is not available in most pathology labs. In this study, we evaluated the applicability of conventional IHC with D5F3 antibody in routine pathological practice and proposed detection methods and procedures that ensure that patients with *ALK*+ are not missed.

**Methods:**

FISH and IHC analyses were performed on 297 lung adenocarcinoma cases. VENTANA IHC and qRT-PCR assay were applied to evaluate *ALK*-fusion status in the discordant cases of FISH and IHC. The association of *ALK*+ with clinicopathological characteristics was statistically analyzed.

**Results:**

IHC had 100% sensitivity and 81.8% specificity for detecting *ALK+*. Eight ALK-expressed cases were *ALK*-, five of which had *ALK* fusion detected by qRT-PCR analysis. Three of these five cases showed ALK expression using VENTANA IHC assay. *ALK+* was associated with younger age and lymph node metastasis in this Chinese lung adenocarcinoma patient cohort.

**Conclusions:**

The advantages of low cost and 100% sensitivity allow conventional IHC to serve as a robust diagnostic tool for screening patients with *ALK*+, especially in pathology labs without a VENTANA IHC platform. For cases in which ALK is weakly expressed, qRT-PCR is necessary as a diagnostic test for *ALK*-fusion detection.

**Virtual slides:**

The virtual slide(s) for this article can be found here: http://www.diagnosticpathology.diagnomx.eu/vs/2269448351088278.

## Introduction

Lung cancer is the most common cause of cancer death worldwide, estimated to be responsible for nearly 1.38 million cancer deaths per year [1]. Despite improvements in the prevention and treatment of lung cancer, the overall 5-year survival rate remains at 15% [2]. Efforts have been made to develop new treatment strategies. In recent years, rearrangements of the anaplastic large cell kinase (*ALK*) gene have been discovered in approximately 5% of lung adenocarcinomas, resulting in the constitutive expression of a fusion protein - most commonly *EML4-ALK* - with oncogenic activity [3-7]. Crizotinib, a potent and specific small molecule inhibitor of both ALK and c-MET tyrosine kinases [8-10], was approved by the Food and Drug Administration (FDA) for the treatment of non-small-cell lung cancer (NSCLC) patients with *ALK* gene rearrangement (*ALK*+).

The FDA-approved Vysis ALK Break Apart FISH Probe Kit (Abbott Molecular) was mandated for *ALK*+ testing in crizotinib trials, which in a sense indicates that FISH analysis has been clinically validated. However, the FISH detection of *ALK* gene rearrangement in routine surgical pathology practice remains impractical due to financial and technical problems. Theoretically, reverse transcriptase-polymerase chain reaction (RT-PCR) is a standard method for determining the fusion genes, but the requirement of fresh frozen tissue samples for extracting RNA has limited its application in clinical practice.

IHC is relatively inexpensive and faster and is performed routinely in most surgical pathology practices. Mutation-specific IHC has been demonstrated as a reliable prescreening test for detecting EGFR mutations in lung adenocarcinoma [11]. Recently, a fully automated VENTANA ALK (D5F3) assay was developed using D5F3 primary antibody (commercialized by Cell Signaling Technology or CST) and VENTANA OptiView DAB detection for use with VENTANA automated platforms. Our group demonstrated that the sensitivity and specificity of the VENTANA ALK assay were 100% and 98%, respectively [12]. The VENTANA ALK (D5F3) IHC assay was approved to detect *ALK* rearrangement in pathology practice in the EU and some Asian countries, including China and Japan. However, the application of the VENTAMA ALK IHC assay requires a VENTANA automated platform, which is not available in most pathology labs. In this study, we applied IHC analysis using CST’s D5F3 antibody to detect *ALK* rearrangement in a Chinese lung adenocarcinoma patient cohort to assess the sensitivity and specificity of IHC analysis. In the third detection method, a qRT-PCR assay (Amoy Diagnostics, Xiamen, China) approved by European Conformity (CE marking) and the China Food and Drug Administration (CFDA), was applied on formalin-fixed paraffin embedded (FFPE) samples to analyze the discordant cases of IHC and FISH.

## Materials and method

### Clinical materials and tissue microarray (TMA) construction

This study included 297 FFPE samples with lung adenocarcinoma diagnosed at the Cancer Institute and Hospital, Chinese Academy of Medical Sciences (CICAMS) in Beijing, between January 2009 and March 2012. Among the 297 cases, 218 were unselected and 79 cases were not effectively treated using conventional treatment.

Among the 218 unselected cases, 178 (with enough tissue) were constructed onto seven TMAs to represent biopsies. A 1.5 mm diameter core was taken from the cancer area based on hematoxylin and eosin (H&E)-stained sections of each sample. The remaining 39 unselected cases (without enough tissue) and 79 selected cases were cut into tissue sections. In the cases where tissue sections/cores fell off the slides during FISH or IHC analysis, tissue sections were re-cut. The collection of these specimens was approved by the National Cancer Center Ethics Committee.

The patients’ medical records were reviewed to obtain their clinicopathological parameters including age at diagnosis, sex, smoking history, tumor size, histological classification and pathological TNM stage.

### IHC

Immunohistochemical staining was performed on 4 μm-thick FFPE tissue sections or TMAs. Briefly, the slides were deparaffinized and antigen retrieval was then performed in a steam cooker for 1.5 minutes in 1 mM EDTA, pH 9.0 (Maixin Biological Techology Co. Ltd., Fuzhou, China). ALK (D5F3) rabbit monoclonal (Cell Signaling Technology, Danvers, MA, USA) was applied at 1:150 in SigalStain antibody diluent (Cell Signaling Technology, Danvers, MA, USA) for 1 h. Universal secondary antibody (DAKO) was applied for 15 min. Diaminobenzidine or 3-amino-9-ethylcarbazole was used as chromogens and slides were counterstained with haematoxylin before mounting.

The criteria for scoring ALK were as follows. First, the intensity was graded as 0, negative; 1, weak (light brown); 2, moderate (brown); and 3, strong (dark brown). Second, the proportion of positive tumor cells was graded: 0, no positive cells; 1, <10%; 2, 11%-30%; 3, 31%-50%; 4, 51-70%; and 5, >70%. A final score was derived by adding the two primary scores. Final scores of 0 were defined as “negative expression” (−); scores of 2–5 as “weakly positive expression” (+); and scores of 6–8 as “strongly positive expression” (++).

Fully automated VENTANA ALK (D5F3) IHC analysis was performed as previously described [12]. According to the manufacture’s scoring algorithm, a binary scoring system (positive or negative for ALK status) was adopted to evaluate the staining results. The presence of strong granular cytoplasmic staining in tumor cells (any percentage of positive tumor cells) was considered to be ALK positive while the absence of strong granular cytoplasmic staining in tumor cells was deemed to be ALK negative.

### FISH

FISH was performed on 3 μm-thick FFPE tumor tissues using a break-apart probe specific to the ALK locus (Vysis LSI ALK Dual Color, Break Apart Rearrangement Probe; Abbott Molecular, Abbott Park, Illinois, USA) according to the manufacturer’s instructions. Tumor cells, the nuclei of which had one or more FISH signals of each color, were enumerated. A positive cell was defined as one in which the nucleus had split signals (two or more signal diameters apart) or a single orange signal (deleted green signal) in addition to fused and/or split signals. A sample was considered positive if >25 cells out of 50 were positive. If a sample had 5 to 25 positive cells (10 to 50%), another 50 tumor cells were counted. If the average percentage of positive cells in 100 tumor cells was <15% (<15/100), the sample was considered negative. If the average percentage of positive cells was ≥15% (≥15/100), the sample was considered positive. TMA cores with high backgrounds or very weak signals that affected the signal assessment were excluded from the analysis.

### Real-time quantitative reverse transcription PCR (qRT-PCR)

The *EML4*-*ALK* fusion mRNA was detected by qRT-PCR using an AmoyDx EML4-ALK Fusion Gene Detection Kit (Amoy Diagnostics, Xiamen, China). Briefly, total RNA was extracted with an AmoyDx FFPE RNA Kit (Spin Column) from 5–10 μm-thick FFPE sections with over 70% tumor cells. For each sample, 100–500 ng of extracted RNA was used for reverse transcription into cDNA at 42°C for 1 h. Real-time PCR was then carried out in each of the four reactions of the *EML4-ALK* Fusion Gene Detection Kit according to the manufacturer’s protocol. Reaction 1 amplifies *EML4-ALK* variants 1, 2, 3a and 3b (variants 1/2/3a/3b); reaction 2 amplifies *EML4-ALK* variants 4 and 4′; reaction 3 amplifies *EML4-ALK* variants 5a, 5b, 5′ and 8 (variants 5a/5b/5′/8); and reaction 4 amplifies the reference gene *beta-actin*. All of the assays were performed on an Agilent Mx3000P QPCR instrument (Agilent Technologies, Santa Clara, CA). The following PCR procedure was used: an initial denaturation at 95°C for 5 min followed by 95°C for 25 s, 64°C for 20 s and 72°C for 20 s to ensure the specificity and 31 cycles of 93°C for 25 s, 60°C for 35 s and 72°C for 20 s to perform the data collection. The quantitative judgment was according to the fusion fluorescence signal. Assay reactions achieving Ct values of ≤30 cycles were considered positive for one of the variants detected by that reaction mixture. A housekeeping gene (*beta-actin*) was used to control the integrity of the RNA.

### Statistical analysis

The statistical analysis of the tumors’ size and age was carried out using Student’s t tests. The values are shown as mean ± SD. The relationship between *ALK*+ and clinicopathological variables was analyzed with the chi-square test. Statistical significance was defined as p < 0.05.

## Results

### Concordance of ALK IHC and FISH

**Figure 1:**
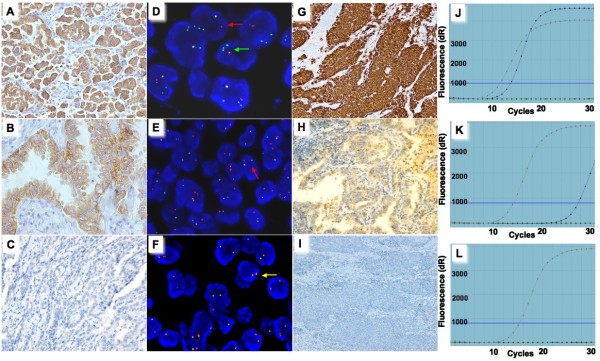
**Representative cases of IHC staining, FISH and qRT-PCR analysis in lung adenocarcinoma. (A-C)** ALK IHC staining using CST’s D5F3 antibody. **(A)** Cytoplasmic reactivity of strong intensity in tumor cells (original magnification, x40). **(B)** Weak to moderate cytoplasmic reactivity in tumor cells (original magnification, x100). **(C)** No staining in tumor cells (original magnification, x200). **(D-F)** FISH analysis using Vysis ALK Break-Apart probes. **(D)** The *ALK*+ case in which the majority of cells contained more than one copy of a single green signal without a corresponding orange signal in addition to fused signals using FISH analysis. Green arrow represents more than one copy of a single green signal, red arrow represents single red or split red-green signals indicative of *ALK*-rearrangement and yellow arrow represents touching red-green signals not indicative of ALK-rearrangement. **(E)***ALK+* case with split red-green signals. **(F)** NSCLC case without *ALK* rearrangement. **(G-I)** VENTANA ALK (D5F3) IHC assay revealed no expression in *ALK*- patients and strong expression in *ALK*+ patients. **(G)** Strong ALK expression (original magnification, x20). **(H)** Unspecific staining (original magnification, x40). **(I)** No ALK expression (original magnification, x20). **(G-L)** Graphs from qRT–PCR showing change in the normalized reporter signal (delta Rn) against PCR cycle number. **(J)***ALK* fusion was detected at around 14 cycles of qRT-PCR analysis in a case with strong ALK expression. **(K)***ALK* fusion was detected at around 28 cycles in a case with weak ALK expression. **(L)** No *ALK* fusion was detected with endogenous control gene, beta-actin, expressed normally.

Using the newly developed antibody, ALK (D5F3), we analyzed ALK expression in 297 lung adenocarcinoma cases. The cases with strongly or weakly positive ALK expression showed readily appreciable cytoplasmic staining (Figures 1A and 1B). In contrast, the cases with negative expression did not show any discernable staining (Figure 1C). Strong ALK expression was identified in 32 cases, weak expression in 12 cases and no expression in 253 cases (Table 1).

**Table 1:** Correlation of IHC and FISH

		**IHC**		**Total**
	**++**^**a**^	**+**^**b**^	**-**^**c**^	
FISH+	31(96.9%)	5(41.7%)	0(0%)	36
FISH-	1(3.1%)	7(58.3%)	242(100%)	250
Total	32	12	242	286

^a^strongly positive ALK expression. ^b^ weakly positive ALK expression. ^c^negative ALK expression.

FISH analysis was performed on the 297 cases to evaluate *ALK* gene rearrangement status. Two hundred and eighty-six out of 297 cases were informative for FISH analysis and 33 cases were identified with *ALK*+ (Figure 1E). Thirty of the 33 *ALK*+ cases showed strong ALK expression and the other 3 showed weak ALK expression. Therefore, there were 11 cases that showed ALK expression but were ALK-. We re-reviewed the FISH slides of the 11 discordant cases (2 cases with strong and 9 cases with weak ALK expression), and 3 cases (1 with strong and 2 with weak ALK expression) were identified as *ALK*+ while 8 (1 case with strong and 7 with weak ALK expression) were still *ALK*- (Table 1, Figure 1F).

Regarding the 3 *ALK*+ cases, which were not identified by the original FISH analysis, a case-by-case analysis revealed the following:

Case 1 The dominant FISH signal pattern in this case was more than one copy of a single green signal without a corresponding orange signal in addition to fused signals (Figure 1D). According to the ALK signal enumeration guide, this indicated a deletion of the orange portion of the ALK probe, which targeted the drug targeting area. Therefore, we initially considered this case as negative. After re-reviewing the FISH analysis, we found there were some areas containing scattered *ALK*+ cells with one or more copies of single green signals in addition to fused signals and a single red signal. The first 50 cells counted revealed 8 *ALK*+ cells. The second and third cell count in another 100 cells by different readers revealed 6 and 7 *ALK*+ cells, respectively. If the first and third 50-cell count was considered, the average percentage of positive cells reached 15%. Therefore, this sample should be considered positive.

Case 1 and 3 For these two cases, originally constructed on TMA and IHC, analysis showed strongly positive staining in one core and weakly positive staining in the other. After re-reviewing the FISH slides, we found that there was indeed a small area of each core with a few cells containing subtle break-apart signals. As cell counts were difficult to perform in small areas containing not many cancer cells, we cut the tissue sections. The IHC analysis still demonstrated strongly and weakly positive ALK expression, respectively. The FISH analysis in the tissue sections showed *ALK*+.

According to the final result of FISH analysis, 36 out of the 286 lung adenocarcinoma cases were identified with *ALK*+. None of IHC negative cases were *ALK*+, demonstrating 100% sensitivity. Eight IHC-positive cases (1 strongly and 7 weakly positive cases) did not show *ALK* gene rearrangement, resulting in 81.8% specificity. The concordance rate of IHC and FISH is 97.2% (Table 1).

### qRT-PCR and VENTANA ALK IHC analysis of discordant cases

To further identify whether eight discordant cases of IHC and FISH carried *ALK* fusion at the RNA level, a qRT-PCR analysis was applied. Positive qRT-PCR results were observed in 5 cases (1 strongly and 4 weakly positive cases) (Table 2). Among the 5 cases, 3 (1 strongly and 2 weakly positive cases) were shown to have ALK expression using VENTANA ALK IHC analysis (Figure 1G). The *ALK* fusion in these 3 cases was detected at around 14 of 30 qRT-PCR cycles (Figure 1J). Regarding the other two cases, although weak staining in cancer cells could be observed (Figure 1H), they were considered negative according to the manufacturer’s scoring algorithm (details in Materials and Method section). The *ALK* fusion in these 2 cases was detected at around 28 of 30 qRT-PCR cycles (Figure 1K). The remaining 3 of the 8 discordant cases showed neither VENTANA ALK staining nor *ALK* fusion (Figures 1I and 1L).

**Table 2:** VENTANA IHC and qRT-PCR analysis of all weakly positive and discordant cases detected by CST ALK (D5F3)

**Sample ID**	**FISH**	**IHC (CST)**	**IHC (VENTANA)**	**qRT-PCR**
				**Variant type**
9	Positive	1+	Positive	*EML4-ALK* variant 1/2/3a/3b
37	Positive	1+	Positive	*EML4-ALK* variant 1/2/3a/3b
67	Positive	1+	Positive	*EML4-ALK* variant 1/2/3a/3b
94	Positive	1+	Positive	*EML4-ALK* variant 1/2/3a/3b
98	Positive	1+	Positive	*EML4-ALK* variant 1/2/3a/3b
28	Negative	2+	Positive	*EML4-ALK* variant 1/2/3a/3b
171	Negative	1+	Positive	*EML4-ALK* variant 1/2/3a/3b
203	Negative	1+	Positive	*EML4-ALK* variant 1/2/3a/3b
21	Negative	1+	Negative	*EML4-ALK* variant 1/2/3a/3b
36	Negative	1+	Negative	*EML4-ALK* variant 1/2/3a/3b
41	Negative	1+	Negative	Negative
39	Negative	1+	Negative	Negative
74	Negative	1+	Negative	Negative

VENTANA ALK IHC and qRT-PCR assays were also applied to the remaining 5 of the 12 ALK weakly expressed cases, which were concordant with FISH analysis. These 5 cases were shown to have ALK expression detected by VENTANA ALK IHC, and ALK fusion revealed by qRT-PCR analysis (Table 2).

### Clinicopathological characteristics of patients with *ALK*+

Using FISH analysis as a standard detection method, the clinicopathological characteristics of the *ALK*+ and *ALK*- patients were compared and the results are shown in Table 3. As the median ages of the positive and negative groups were 48 and 58 years, respectively, the *ALK+* patients were significantly younger (p <0.001). Patients with *ALK+* were more likely to have lymph node metastasis compared to *ALK*- patients (p = 0.002). No correlation was observed between *ALK+* and *ALK*- cases in terms of sex, smoking habit, tumor size, pT, M factors or pathologic TNM stage.

**Table 3:** Clinicopathologic comparisons between EML4–ALK fusion-positive and fusion-negative lung adenocarcinomas

	**Overall**	**EML4–ALK(+)**	**EML4–ALK(−)**	
	**n = 287**	**n = 37 (%)**	**n = 250 (%)**	***P *** **value**
Age	Mean ± SD	48.16 ± 11.529	58.17 ± 10.03	<0.001
	Median	48	58	
	Range	24-70	25-81	
Sex	Male	20(13.1)	133(86.9)	0.999
	Female	17(13.1)	113(86.9)	
	Nonavailable	0	4	
Smoking	Never smoker	22(13.8)	138(86.2)	0.239
	Ever smoker	9(8.9)	92(91.1)	
	Nonavailable	6	20	
Tumor size(mm)	Mean ± SD	38.42 ± 24.263	37.59 ± 16.837	0.872
	Median	32.50	35.00	
	Range	7-100	10-90	
pT status	pT1	4(13.8)	25(86.2)	0.350
	pT2	14(8.3)	154(91.7)	
	pT3-T4	5(15.6)	27(84.4)	
	Nonavailable	14	44	
pN status	pN0	2(2.0)	100(98.0)	0.002
	pN1	11(16.7)	55(83.3)	
	pN2-3	7(13.5)	45(86.5)	
	Nonavailable	17	50	
pTNM stages	pStage I	2(2.5)	79(97.5)	0.028
	pStage II	9(12.2)	65(87.8)	
	pStage III	9(14.3)	54(85.7)	
	Nonavailable	17	52	

## Discussion

In this study, we applied IHC and FISH analyses using CST’s D5F3 antibody in a Chinese lung adenocarcinoma sample cohort. An accurate FISH analysis depends on multiple factors including fine equipment, skilled personnel, well-preserved FFPE samples, enough cancer cells, etc. In this study, two cores in TMAs were not identified with *ALK*+ in initial FISH analysis due to a lack of cancer cells. Similarly, in biopsies, the numbers of cancer cells is often very limited, making an accurate FISH analysis difficult. With the IHC analysis in this study, almost all of the cancer cells in the two cores showed ALK expression, despite the fact that only a few *ALK*+ cells were revealed by FISH analysis. A <100% rate of cellular positivity in *ALK*+ tumors has been demonstrated to be due to the technical limitations of FISH analysis [13]. Therefore, combining IHC and FISH analyses results in ALK status being more accurately evaluated in biopsies.

IHC analysis using CST’s D5F3 antibody has been demonstrated with 100% sensitivity [12,14-16], suggesting that IHC analysis is an effective way to prescreen patients for FISH analysis in the clinical diagnosis process [14,15,17]. For IHC negative cases, FISH analysis is not necessary. In strongly positive IHC cases, FISH analysis also may not be necessary. Although there was one strongly positive IHC case, which was shown with *ALK*- by FISH analysis, the VENTANA ALK assay and qRT-PCR analysis revealed ALK expression and *ALK* fusion, respectively. In addition, it has been reported that the lung cancer patient with IHC-positive and FISH-negative ALK had a dramatic response to crizotinib [18]. Therefore, the patient in our case may benefit from crizotinib.

Weakly positive IHC cases must be carefully examined. In this study, 7 out of 12 (58.3%) weakly positive cases were discordant with FISH analysis. Using the VENTANA ALK IHC assay, three out of the seven weakly positive cases showed ALK expression and could be treated with crizotinib. Using qRT-PCR analysis, five out of the seven weakly positive cases showed *ALK* fusion at the RNA level. Therefore, there were two cases in which the qRT-PCR analysis result was discordant with the VENTANA ALK IHC assay. Compared to negatively expressed ALK cases without any staining (Figure 1I), these two cases were indeed weakly stained in cancer cells using the VENTANA ALK IHC analysis (Figure 1H). However, according to the VENTANA ALK IHC assay scoring algorithm, the weak staining in these two cases was regarded as unspecific and thus considered negative. Although qRT-PCR analysis demonstrated *ALK* fusion in these two cases, it was detected in a very late stage of the qRT-PCR process. We speculated that the percentage of tumor cells with *ALK* fusion might be very low in these two cases. However, with very high sensitivity (1 in 100 DNA), they would still be detected by qRT-PCR analysis. Whether these two patients would benefit from crizotinib was difficult to predict, as no relevant study has been reported. Further study is required.

Previous reports have shown that *ALK*+ lung cancers are characterized by younger patients, non-smokers or light smokers when compared with *ALK*- patients [6,7,19-23]. In this study, the *ALK*+ patients were significantly younger and more likely to have lymph node metastasis compared to *ALK*- patients. However, *ALK*+ and *ALK*- lung adenocarcinomas showed no difference in sex, smoking habit, tumor size, pT, M factors or pathologic TNM stage. The screening was limited in this study to the lung adenocarcinomas of Chinese patients. There may be an underlying difference in the subject population by race and clinical characteristics.

In conclusion, with advantages such as a low cost and 100% sensitivity, IHC with CST’s D5F3 antibody can serve as a robust diagnostic tool with which to routinely screen lung adenocarcinoma patients with *ALK*+ in pathology labs that do not have access to VENTANA automated IHC platforms. For weakly expressed ALK cases, qRT-PCR analysis, especially when applied on FFPE samples, is suggested as a diagnostic test for *ALK* fusion detection.

## Competing interest

The author’s declared that they have no competing interest.

## Authors’ contributions

Study concept and design: DL, LS, JY. Analysis and interpretation of data: LS, FL, LG, XY. Drafting of the manuscript: LS, FL, DL. All authors have read and approved the final manuscript.
